# Diabetes related risk factors did not explain the increased risk for urinary incontinence among women with diabetes. The Norwegian HUNT/EPINCONT study

**DOI:** 10.1186/1471-2490-9-11

**Published:** 2009-09-10

**Authors:** Marit Helen Ebbesen, Yngvild S Hannestad, Kristian Midthjell, Steinar Hunskaar

**Affiliations:** 1 Section for General Practice, Department of Public and Primary Health Care, University of Bergen, Norway; 2 Department of Obstetrics and Gynecology, Haukeland University Hospital, Bergen, Norway; 3 HUNT Research Centre, Department of Public Health and General Practice, Norwegian University of Science and Technology (NTNU), Verdal, Norway

## Abstract

**Background:**

Previous studies have shown an association between diabetes mellitus (DM) and urinary incontinence (UI) in women, especially severe UI. The purpose of this study was to investigate whether diabetes related variables could explain this association.

**Methods:**

The study is part of the EPINCONT study, which is based on the large Nord-Trøndelag Health Study 2 (HUNT 2), performed in the county of Nord-Trøndelag, Norway, during the years 1995 - 1997. Questions on diabetes and UI were answered by a total of 21 057 women aged 20 years and older. Of these 685 were identified as having diabetes, and thus comprise the population of our study. A variety of clinical and biochemical variables were recorded from the participants.

**Results:**

Blood-glucose, HbA1c, albumine:creatinine ratio (ACR), duration of diabetes, diabetes treatment, type of diabetes, cholesterol and triglycerides did not significantly differ in women with and without UI in crude analyses. However, the diabetic women with UI had more hospitalizations during the last 12 months, more homecare, and a higher prevalence of angina and use of oestrogene treatment (both local and oral/patch). After adjusting for age, BMI, parity and smoking, there were statistically significant associations between any UI and angina (OR 1.89; 95% CI: 1.22 - 2.93), homecare (OR 1.72; 95% CI: 1.02 - 2.89), and hospitalization during the last 12 months (OR 1.67; 95% CI: 1.18 - 2.38). In adjusted analyses severe UI was also significantly associated with the same variables, and also with diabetes drug treatment (OR 2.10; 95% CI: 1.07 - 4.10) and stroke (OR 2.47; 95% CI: 1.09 - 5.59).

**Conclusion:**

No single diabetes related risk factor seems to explain the increased risk for UI among women with diabetes. However, we found associations between UI and some clinical correlates of diabetes.

## Background

Recent studies have established that urinary incontinence (UI) is more prevalent among women with diabetes [1,2], the UI also seems to be of a different type spectrum [3] and to be more severe [1,4]. The association between diabetes and UI has partly been explained by differences in non-diabetic factors like age, body mass index (BMI) and obesity, but still seems to be statistically significant after adjustment for such factors [5]. Several direct diabetes related factors could possibly lead to an increased risk for UI in women with diabetes; examples are hyperglycemia induced polyuria and damage to nerves and blood vessels [6,7]. A recently published study of patients with type 2 diabetes, found that the threshold value for women to develop bladder dysfunction was HbA1c >7% [8].

In a previous paper with data from the EPINCONT study we found a statistically significant association between diabetes and UI, with an OR of 1.83 (95% CI: 1.57-2.15) [5]. The association was still significant after adjusting for age, BMI, parity and smoking (OR 1.21, 95% CI: 1.01-1.44). Using the same data set we now present analyses on how specific factors related to the diabetes disease and its management may further explain this association between diabetes and UI.

## Methods

The Nord-Trøndelag Health Survey 2 (HUNT 2) was a cross-sectional survey conducted in the county of Nord-Trøndelag, Norway, in 1995-97. It was a follow-up study of a similar survey, HUNT 1, conducted in 1984-86. Nord-Trøndelag is well suited for surveys like this since its geography, demography and inhabitants are fairly representative for Norway. Nord-Trøndelag, however, has no big cities, and its inhabitants have an income and educational level slightly below average.

All the inhabitants aged 20 years or older in the county were invited to participate (n = 94 197), 47 313 were women. The invitations mailed to the inhabitants included questionnaire 1 (Q1), which was answered by 34 755 women. The participants were asked to bring the completed questionnaire to a screening station, where among other measurements blood pressure, height and weight were measured, and blood samples were drawn. The women who met at the screening station received questionnaire 2 (Q2), which was different from men, and which among other topics contained questions on UI.

The incontinence part of the questionnaire is known as the EPINCONT study (Epidemiology of incontinence in the county of Nord-Trøndelag) [9]. Urinary incontinence was defined in accordance with the standards of the International Continence Society as "any leakage of urine" [10]. Type of incontinence was determined by asking the women about the situations in which they experienced leakage. If leakage occurred in association with lifting heavy items, laughing, coughing or sneezing the women were categorised as having symptoms of stress UI. Leakage in association with a strong urge to void was categorised as urge UI. The incontinent women who had both stress and urge symptoms were classified as having mixed type incontinence. For a small number of women (19) type could not be determined due to incomplete answers [9]. The women further answered questions about frequency of leakage (four levels), and amount of leakage (three levels), which were used to determine Sandvik's severity index [11]

Q1 also had a question on diabetes status, and those answering affirmative to the question "Do you have or have you had diabetes?" received questionnaire 3 (Q3) containing further questions on their diabetes situation [12]. This questionnaire contained questions on subjects like diagnosis, treatment (insulin, tablets, none), monitoring of the diabetes (home monitoring, monitored by health personal), diet, membership in Norwegian Diabetes Association, vision issues, hospitalization since diagnosis, quality of life and foot problems. The participants identified as having diabetes had an additional 5 ml EDTA blood drawn for HbA1c analysis. They also received three urine tubes for overnight urine sampling, which were analysed for microalbuminuria and creatinine to calculate the albumine:creatinine ratio (ACR). Women who through Q1 were identified as having diabetes were given new appointments for fasting serum sample, used for analyses of glucose, C-peptide and anti-GAD. Anti-GAD was analysed via immunoprecipitation, using (3H)leucine translation-labelled GAD65 as an indicator. The women were cathegorized as having type II DM if concentration of anti-GAD was below 0.08. Further information about methods and instruments used for blood-sample analyses are described elsewhere [12].

Response rate was high, 80% (27 936) of the women meeting at the screening stations answered the incontinence part of the study, and out of these 75% answered the questions on diabetes. This provided us with data on a total of 685 women with diabetes.

### Statistics

Data were analysed using SPSS (version 15.0). Statistical significance was accepted at a 5% level (p < 0.05). We used Chi-Squared Test to test for differences in prevalence of diabetes type, non fasting glucose, HbA1c, duration of diabetes, diabetes treatment, cholesterol value, triglyceride value, albumine: creatinine ratio, oestrogene use, angina, heart attack, stroke or cerebral hemorrhage, hospitalization during the last 12 months and homecare between women with and without UI. Logistic regression analyses were performed to investigate the association between diabetes-related factors and UI among the women with diabetes. In a previous study we found that age, BMI, parity and smoking confounded the association between diabetes and UI, and these factors were therefore adjusted for in the final logistic regression analyses also in the current study [5]. Analyses were performed separately for each of the different outcomes under investigation, continent women serving as reference group in all the analyses. Parity and smoking were categorical variables, and no children and never smoked were used as alternative outcomes. Age and BMI were used as continuous variables. Odds ratios with corresponding 95% confidence intervals (CI) were effect measures. Duration of diabetes was used as a continuous variable. We chose to use the following variables as categorical, with the following alternative outcomes: blood glucose < 11.1 mmol/l, HbA1c ≤ 7.0 mmol/l, ACR < 2.5 mg/mmol, cholesterol < 7.7, triglyceride ≤ 2.45, diet treatment, no hospitalization during the last 12 months, no homecare, no angina, no stroke and no heart attack.

**Figure 1:**
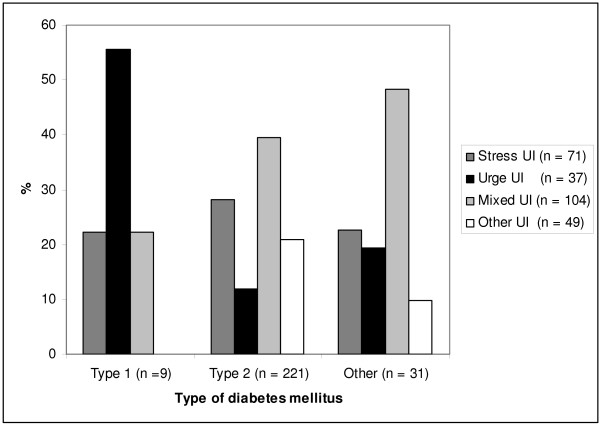
**Distribution of UI type by DM type**.

The women with diabetes categorized into a different type than type 1 DM and type 2 DM, and those not possible to categorize due to incomplete data, were added together into the group "other type of DM" for our analyses in figure 1.

### Ethics

The main study had ethical recommendation from both the Regional and National Ethics Review Boards. Attendance was completely voluntary and the subjects gave an extensive written consent to the use of the collected material. The survey was also approved by the Norwegian Data Inspectorate.

## Results

Table 1 contains data with respect to several diabetes related factors for women with and without UI. We found only a few statistically significant associations between such factors and UI when performing Chi square tests. Angina pectoris was significantly more prevalent in the UI group, and there was also a statistically significant difference in oestrogene use between the two groups, oestrogene use being less prevalent among the incontinent women. In the UI group a larger proportion had been hospitalized during the last 12 months, and these women also received more home care services.

**Table 1:** Distribution of diabetes related variables and general health status variables in diabetic women with and without UI (n)

**Variable**	**Women without UI** **N = 418 (61%)**	**Women with UI** **N = 267 (39%)**	**P - value**
**Type diabetes (514)**			0.167
Type 1 DM	23 (7.2)	8 (4.1)	
Type 2 DM	244 (76.5)	162 (83.1)	
Other	52 (16.3)	25 (12.8)	
**Non-fasting glucose (683)**			0.403
< 11.1 mmol/	310(74.3)	191 (71.8)	
≥ 11.1 mmol/l	107(25.7)	75 (28.2)	
**HbA1c (n = 659)**			0.468
≤ 7.0 mmol/l	141 (34.8)	90 (35.4)	
> 7.0 mmol/l	264 (65.2)	164 (64.6)	
**Duration of diabetes (506)**			0.364
0 - 10 years	216 (68.6)	123 (64.4)	
11 - 20 years	71 (22.5)	55 (28.8)	
21 - 30 years	20 (6.3)	8 (4.2)	
≥ 31 years	8 (2.5)	5 (2.6)	
**Diabetes treatment (510)**			0.542
Drugs	213 (67.8)	138 (70.4)	
Diet only	101 (32.2)	58 (29.6)	
**Cholesterol (683)**			0.906
< 7.7 mmol/l	345 (82.7)	221 (83.1)	
≥ 7.7 mmol/l	72 (17.3)	45 (16.9)	
**Triglycerides (683)**			0.855
≤ 2.45 mmol/l	260 (62.4)	164 (61.7)	
> 2.45 mmol/l	157 (37.6)	102 (38.3)	
**ACR (617)**			0.489
< 2.5 mmol/l	309 (81.7)	190 (79.5)	
≥ 2.5 mmol/l	69 (18.3)	49 (20.5)	
**Estrogene use (oral or patch) (492)**			0.012
Using now	29 (9.3)	30 (16.8)	
Used before	16 (5.1)	15 (8.4)	
Never used	268 (85.6)	134 (74.9)	
**Estrogene use (local) (455)**			< 0.001
Using now	30 (10.6)	33 (19.3)	
Used before	12 (4.2)	19 (11.1)	
Never used	242 (85.2)	119 (69.6)	
**Angina (668)**			< 0.001
No	347 (85.0)	190 (73.1)	
Yes	61 (15.0)	70 (26.9)	
**Heart attack (671)**			0.094
No	385 (93.9)	236 (90.4)	
Yes	25 (6.1)	25 (9.6)	
**Stroke (663)**			0.072
No	385 (95.3)	238 (91.9)	
Yes	19 (4.7)	21 (8.1)	
**Hospitalization during last 12 months (653)**			0.001
No	232 (58.1)	114 (44.9)	
Yes	167 (41.9)	140 (55.1)	
**Home care (373)**			< 0.001
No	146 (69.9)	82 (50.0)	
Yes	63 (30.1)	82 (50.0)	

Figure 1 shows the distribution of types of UI by types of DM. Mixed UI was the most common type of UI among women with type 2 DM and other types of DM, urge UI was the most common type in women with type 1 DM. When using Chi square tests to test for differences in type distribution, the only statistically significant difference was between type 1 and type 2 diabetes (p = 0.02). Table 2, 3 and 4 show data from logistic regression analyses. Table 2 contains analyses for any UI and severe UI, whilst table 3 and 4 contains analyses by type of UI. Table 3 presents unadjusted analyses, whilst table 4 contains the adjusted analyses. All analyses were adjusted for age, BMI, parity and smoking. In adjusted analyses any UI was associated with angina, home care and hospitalization during the last 12 months, but not with more direct clinical related diabetes factors. Severe UI was associated with the same factors, but in addition also with stroke. Antidiabetic drug treatment was associated with severe UI only. In adjusted analyses by UI types the only statistically significant associations were between mixed UI and angina, and between mixed UI and hospitalizations during the last 12 months. No associations between diabetes management related factors and type of UI were found.

**Table 2:** Logistic regression analyses of UI severity (any or severe) and diabetes related variables

**Variable**	**Any UI** **N = 267**		**Severe UI** **N = 117**	
	
	**Unadjusted**	**Adjusted †**	**Unadjusted**	**Adjusted †**
**Glucose**				
< 11.1 mmol/l	1.0	1.0	1.0	1.0
≥ 11.1 mmol/l	1.14 (0.81 - 1.61)	0.97 (0.66 - 1.42)	1.77 (1.15 - 2.73)*	1.53 (0.94 - 2.47)
**HbA1c**				
≤ 7.0 mmol/l	1.0	1.0	1.0	1.0
> 7.0 mmol/l	0.97 (0.70 - 1.35)	0.92 (0.64 - 1.34)	1.49 (0.94 - 2.37)	1.45 (0.85 - 2.47)
**Duration of diabetes**	1.01 (0.99 - 1.03)	1.00 (0.98 - 1.03)	1.01 (0.98 - 1.04)	1.00 (0.97 - 1.04)
**Diabetes treatment**				
Diet only	1.0	1.0	1.0	1.0
Drugs (insulin or tablets)	1.13 (0.77 - 1.66)	1.0 (0.65 - 1.55)	1.94 (1.09 - 3.47)*	2.10 (1.07 - 4.10)*
**ACR**				
< 2.5 mmol/l	1.0	1.0	1.0	1.0
≥ 2.5 mmol/l	1.16 (0.77 - 1.74)	1.02 (0.65 - 1.61)	1.33 (0.78 - 2.26)	1.00 (0.55 - 1.85)
**Triglycerides**				
≤ 2.45 mmol/l	1.0	1.0	1.0	1.0
> 2.45 mmol/l	1.03 (0.75 - 1.41)	0.79 (0.56 - 1.13)	1.24 (0.82 - 1.88)	1.09 (0.69 - 1.73)
**Cholesterol**				
< 7.7 mmol/l	1.0	1.0	1.0	1.0
≥ 7.7 mmol/l	0.98 (0.65 - 1.47)	0.91 (0.59 - 1.43)	1.06 (0.62 - 1.80)	0.91 (0.50 - 1.65)
**Angina**				
No	1.0	1.0	1.0	1.0
Yes	2.10 (1.42 - 3.08)***	1.89 (1.22 - 2.93)**	2.36 (1.48 - 3.79)***	2.05 (1.20 - 3.48)**
**Heart attack**				
No	1.0	1.0	1.0	1.0
Yes	1.63 (0.92 - 2.91)	1.31 (0.68 - 2.54)	2.22 (1.11 - 4.42)*	1.69 (0.76 - 3.75)
**Stroke**				
No	1.0	1.0	1.0	1.0
Yes	1.79 (0.94 - 3.40)	1.84 (0.91 - 3.73)	2.97 (1.44 - 6.13)**	2.47 (1.09 - 5.59)*
**Home care**				
No	1.0	1.0	1.0	1.0
Yes	2.32 (1.51 - 3.55)***	1.72 (1.02 - 2.89)*	3.57 (2.12 - 6.00)***	2.80 (1.48 - 5.29)**
**Hospitalization during last 12 months**				
No	1.0	1.0	1.0	1.0
Yes	1.71 (1.24 - 2.34)**	1.67 (1.18 - 2.38)**	2.41 (1.57 - 3.71)***	2.55 (1.56 - 4.16)***

† = adjusted for age, BMI, parity and smoking. * = p < 0.05 ** = p < 0.01 *** = p < 0.001

**Table 3:** Unadjusted logistic regression analyses of UI by type and diabetes related variables in women with diabetes mellitus.

**Variable**	**Stress UI‡** **N = 77**	**Urge UI‡** **N = 44**	**Mixed UI‡** **N = 127**
**Glucose**			
< 11.1 mmol/l	1.0	1.0	1.0
≥ 11.1 mmol/l	0.96 (0.55 - 1.69)	1.57 (0.81 - 3.06)	1.30 (0.84 - 2.01)
**HbA1c**			
≤ 7.0 mmol/l	1.0	1.0	1.0
> 7.0 mmol/l	0.72 (0.44 - 1.19)	1.97 (0.92 - 4.23)	0.96 (0.63 - 1.47)
**Duration of diabetes**	0.97 (0.93 - 1.01)	1.03 (1.00 - 1.07)	1.02 (0.99 - 1.04)
**Diabetes treatment**			
Diet only	1.0	1.0	1.0
Drugs	0.88 (0.48 - 1.61)	1.99 (0.79 - 4.99)	1.18 (0.71 - 1.93)
**ACR**			
< 2.5 mmol/l	1.0	1.0	1.0
≥ 2.5 mmol/l	0.84 (0.42 - 1.68)	1.22 (0.56 - 2.67)	1.17 (0.70 - 1.96)
**Triglycerid**			
≤ 2.45 mmol/l	1.0	1.0	1.0
> 2.45 mmol/l	0.80 (0.48 - 1.33)	1.19 (0.63 - 2.25)	1.11 (0.74 - 1.67)
**Cholesterol**			
< 7.7 mmol/l	1.0	1.0	1.0
≥ 7.7 mmol/l	0.78 (0.39 - 1.54)	0.61 (0.23 - 1.61)	0.98 (0.58 - 1.65)
**Angina**			
No	1.0	1.0	1.0
Yes	1.04 (0.53 - 2.04)	1.25 (0.55 - 2.82)	2.56 (1.62 - 4.04)***
**Heart attack**			
No	1.0	1.0	1.0
Yes	1.28 (0.51 - 3.21)	0.35 (0.05 - 2.67)	2.11 (1.10 - 4.05)*
**Stroke**			
No	1.0	1.0	1.0
Yes	1.15 (0.38 - 3.48)	2.58 (0.92 - 7.26)	1.89 (0.88 - 4.06)
**Home care**			
No	1.0	1.0	1.0
Yes	2.27 (1.16 - 4.44)*	2.44 (1.11 - 5.33)*	2.22 (1.31 - 3.76)**
**Hospitalization during last 12 months**			
No	1.0	1.0	1.0
Yes	1.38 (0.84 - 2.25)	1.22 (0.64 - 2.30)	1.85 (1.22 - 2.80)**

* = p < 0.05 ** = p < 0.01 *** = p < 0.001

‡ = There is a small discrepancy in number of women in the *any UI *group and *UI type *groups because type could not be determined in all of the women with UI due to incomplete answers.

**Table 4:** Adjusted (†) logistic regression analyses of UI by type and diabetes related variables in women with diabetes mellitus.

**Variable**	**Stress UI‡** **N = 77**	**Urge UI‡** **N = 44**	**Mixed UI‡** **N = 127**
**Glucose**			
< 11.1 mmol/l	1.0	1.0	1.0
≥ 11.1 mmol/l	0.84 (0.45 - 1.56)	1.17 (0.53 - 2.60)	1.17 (0.71 - 1.86)
**HbA1c**			
≤ 7.0 mmol/l	1.0	1.0	1.0
> 7.0 mmol/l	0.69 (0.40 - 1.20)	1.79 (0.74 - 4.35)	0.90 (0.55 - 1.47)
**Duration of diabetes**	0.95 (0.91 - 1.00)	1.03 (0.99 - 1.08)	1.02 (0.99 - 1.05)
**Diabetes treatment**			
Diet only	1.0	1.0	1.0
Drugs	0.77 (0.40 - 1.50)	2.67 (0.87 - 8.25)	1.01 (0.57 - 1.78)
**ACR**			
< 2.5 mmol/l	1.0	1.0	1.0
≥ 2.5 mmol/l	0.66 (0.30 - 1.48)	1.24 (0.50 - 3.09)	1.07 (0.60 - 1.93)
**Triglycerid**			
≤ 2.45 mmol/l	1.0	1.0	1.0
> 2.45 mmol/l	0.61 (0.34 - 1.09)	1.12 (0.53 - 2.34)	0.83 (0.53 - 1.32)
**Cholesterol**			
< 7.7 mmol/l	1.0	1.0	1.0
≥ 7.7 mmol/l	0.87 (0.43 - 1.78)	0.59 (0.20 - 1.75)	0.80 (0.44 - 1.46)
**Angina**			
No	1.0	1.0	1.0
Yes	1.22 (0.58 - 2.55)	0.92 (0.33 - 2.59)	2.25 (1.34 - 3.77)**
**Heart attack**			
No	1.0	1.0	1.0
Yes	1.03 (0.34 - 3.13)	NA	1.61 (0.75 - 3.45)
**Stroke**			
No	1.0	1.0	1.0
Yes	1.33 (0.42 - 4.18)	1.78 (0.47 - 6.71)	1.89 (0.82 - 4.36)
**Home care**			
No	1.0	1.0	1.0
Yes	1.78 (0.76 - 4.14)	2.09 (0.79 - 5.54)	1.68 (0.89 - 3.17)
**Hospitalization during last 12 months**			
No	1.0	1.0	1.0
Yes	1.33 (0.77 - 2.30)	1.44 (0.69 - 3.03)	1.74 (1.10 - 2.78)*

* = p < 0.05 ** = p < 0.01 *** = p < 0.001 NA = Not applicable due to no cases

† = Adjusted for age, BMI, parity and smoking

‡ = There is a small discrepancy in number of women in the *any UI *group *UI type *groups because type of UI could not be determined in all of the women with UI due to incomplete answers.

## Discussion

By analyses of this data set we were not able to explain how specific factors related to the diabetes disease and its management link diabetes to UI. We found some associations between diabetes management and complications and UI, but the biological and laboratory parameters do not seem to explain the association previously documented.

The large cross sectional HUNT study had a high response rate [9]. The UI and DM parts of the study have been validated [11,13,14]. Response rates to the different diabetes variables varied from 45.5% (health care at home), to 99.7% (glucose, cholesterol and triglycerides), and most were above 90%. The response rates for individual variables were similar in women with and without diabetes, so this should not bias the results. The limited number of women in some of the analyses might have contributed to possible type II errors, but differences in prevalences between the group with UI and the group without UI were small. The estimated associations between elevated HbA1c and severe UI (OR 1.45; 95% CI: 0.85 - 2.47) and between elevated HbA1c and urge UI (OR 1.79; 95% CI: 0.74 - 4.35) were marked but not statistically significant, probably due to power limitations. There are therefore indications in our study that some real connections between UI and diabetes measurements may indeed be present.

Previous studies have found correlations between diabetes and UI [1,2,5]. Intensive lifestyle intervention was found to reduce the risk of developing DM in women with increased fasting levels of glucose, and also to reduce incidence of UI compared to a metformin treated group and a placebo treated group [15]. The authors showed that reduced weight in the lifestyle intervention group explained most of the effect, and that direct DM related factors played a minor role for the effect on UI.

That HbA1c (> 7 mmol/l) was not associated with an increased risk for UI in women with diabetes, is in accordance with findings from a recent study on bladder dysfunctions in type 2 diabetes patients [8], also stating, however, that HbA1c was positively correlated with an increase in volume at first desire to void, and mean HbA1c was found to be higher in women with post-void residual volume > 100 ml.

Diabetes complications increase in prevalence with the duration of diabetes. One would therefore expect a correlation between diabetes duration and UI. Assuming that UI could be caused by alteration of the detrusor muscle, the innervation or function of the neuronal component or urothelial dysfunction [16], high levels of glucose over time could be partially responsible for such damage. However, we found no such significant association between duration, blood glucose, HbA1c and UI in our study, and adjustments for age did not change this. But the significant correlation between medical treatment for DM (hence more serious DM) and severe UI, may anyhow indicate that clinical complications may have an impact on the risk of UI after longstanding disease.

An increased rate of hospitalization during the last 12 months indicates that women with DM and UI had a worse health status than women without UI. In our previous study from the same data set we found that women with diabetes had significantly higher prevalence of stroke, heart attack and elevated blood pressure [5]. In the present analysis the diabetic women with UI had significantly more angina than the women without UI, indicating a higher prevalence of cardiovascular problems in the diabetic women with UI. If such disease mechanisms also occur in other organs, inadequate vascularisation of the bladder might contribute to a higher prevalence of UI in women with diabetes. Microvascular changes may damage the innervation of the bladder, alter detrusor muscle function or cause impaired bladder sensation as well as overflow incontinence [17]. Poor health status in elderly patients with diabetes is common. Women with diabetes have higher rates of many chronic diseases, like stroke, kidney disease and chronic heart failure, are less likely to rate their health as good or excellent and they report greater functional disability [18]. Poorly regulated diabetes is shown to be an important determinant for developing diabetic autonome neuropathy, and autonomic function has been shown to be more abnormal in insulin-dependent diabetic patients with coronary heart disease than those without [19]. We are fully aware of, however, that our cross sectional study design cannot establish causality between risk factors and outcomes.

The nonsignificant findings of our study could be due to lack of relevant variables in order to determine an actual connection between DM and UI. If a causal mechanism by neuropathy or microvascular changes is the explanation, we would need more relevant variables including detailed bladder function tests to identify damages caused by DM, and also more sophisticated ways of estimating neuropathy. Kaplan et al emphasize the importance of urodynamic studies in diagnosing voiding dysfunction in diabetic patients, especially to separate UI, cancer or urinary tract infections from diabetic cystopathy [20]. Clinical examinations are costly and difficult to perform as part of epidemiological studies, due to a large number of participants needed, and it is also likely that it would be more difficult to recruit participants to clinical examinations in this field. Future studies should include questions that could possibly replace physical examinations.

The strongest association between diabetes and UI presented in our previous publication was between diabetes and severe UI [5]. The small number of women with severe UI might partly explain why no significant associations were found between the diabetes related factors and severe UI. Only 43 women with severe UI had blood glucose >11.1 mmol/l which might explain why the association after adjustments no longer was significant.

Studies on rats with induced diabetes have found several possible explanations for bladder problems due to diabetes. Paro et al demonstrated a doubling of the threshold volume required to initiate the micturition reflex in rats with diabetes for 8 months [21]. They also found changes in pelvic and hypogastric nerves. Treatment with gangliosides within 4 months could to a large extent reverse these damages. Another study showed that diabetes led to up-regulation of muscarine-acetylcholine receptors in the urinary bladder, which could lead to increased reactivity to acetylcholine and result in detrusor instability [22]. Even if diabetes causes the same changes in the human bladder, epidemiological studies are probably not the best study design to study such connections. This may explain why our previous epidemiological study found a higher prevalence of UI [5], while no diabetes related risk factors in the present study seem to be able to explain the findings.

## Conclusion

No single diabetes related risk factor seems to explain the increased risk for UI among women with diabetes. However, we found associations between UI and some clinical correlates of diabetes. Further epidemiological studies may not be the best strategy to study the mechanisms of increased risk of UI among women with DM.

## Abbreviations

ACR: Albumine: creatinine ratio; BMI: Body mass index; CI: Confidence interval; DM: Diabetes mellitus; EPINCONT: Epidemiology of urinary incontinence in the county of Nord-Trøndelag; HUNT: Nord-Trøndelag Health Study; OR: Odds ratio; UI: Urinary incontinence.

## Competing interests

The authors declare that they have no competing interests.

## Authors' contributions

All authors designed and planned the study. MHE performed the initial analyses and drafted the first manuscript, all authors contributed to the final manuscript. All authors have approved the final manuscript.

## Pre-publication history

The pre-publication history for this paper can be accessed here:


